# A Rare Case of a Third Trimester Intra-abdominal Pregnancy En Caul Delivered via Cesarean Section

**DOI:** 10.7759/cureus.82582

**Published:** 2025-04-19

**Authors:** Reinaldo E Claudio, Thomas Fredericks, Smita Sharma, Mauricio Hernandez, Sindhu Kumar

**Affiliations:** 1 Radiology, University of Florida College of Medicine – Jacksonville, Jacksonville, USA

**Keywords:** cornual rupture, ct in pregnancy, en caul, intra-abdominal pregnancy, spontaneous uterine rupture, third trimester rupture

## Abstract

Uterine rupture is among the most dangerous complications that can occur in pregnancy. The risk of this complication increases with multiparity, prior rupture, and prior cesarean sections. In combination, these pose a serious risk to both patient and fetus and should be properly evaluated in the setting of refractory abdominal pain in pregnancy. We present a case of a 30-year-old pregnant woman at approximately 30 weeks of gestation with a past medical history (PMH) of prior ectopic pregnancies, prior pelvic surgeries, and limited prenatal care who arrived at our institution following reports of an intra-abdominal pregnancy with no fetal heart tones detected on an ultrasound performed at an outside institution. She arrived hemodynamically stable; however, it was notable that she had a critically low hemoglobin of 6.5 and leukocytosis. She received a CT abdomen and pelvis, which demonstrated an intra-abdominal pregnancy with an adjacent fundal defect and prominent hemoperitoneum. The patient was subsequently transported to the operating room and underwent an exploratory laparotomy, with visualization and delivery of a nonviable female infant within the amniotic sac. The intraoperative evaluation of the uterus demonstrated a prominent cornual defect with active bleeding. The decision was made to perform a hysterectomy, and the patient recovered well postoperatively without complications. This case illustrates how a comprehensive evaluation of a pregnant patient, with both lab work and appropriate cross-sectional imaging, can provide critical information regarding obstetrical emergencies such as uterine rupture and highlights a rare finding in the delivery of an intra-abdominal fetus en caul*.*

## Introduction

Uterine rupture during pregnancy is a dangerous obstetric complication associated with significant maternal and fetal morbidity and mortality [1]. Among the highest risk factors for uterine rupture are myometrial scars from prior surgeries [2], often prior cesarean sections, as well as multiparous women, due to serial stretching of the uterine wall [1]. Uterine ruptures most often occur in the third trimester, during labor, and have varying fetal and maternal morbidity depending upon the location of the rupture. Lateral wall ruptures have demonstrated worse outcomes in comparison to midline uterine ruptures [1,3]. Given the relative rarity of uterine rupture, there is scant literature regarding severe obstetrical complications, such as migration of the fetus into the abdominal cavity [4-6]. The diagnosis of uterine rupture is often made via clinical assessment of both mother and fetus, assisted by the use of radiographic imaging as clinically indicated [7,8].

## Case presentation

Here, we report the case of a preterm spontaneous uterine rupture, with complete navigation of the fetus "en caul" to the abdominal cavity. Our patient is a 30-year-old female G10P3144 with a past medical history notable for multiple ectopic pregnancies, previous left salpingectomy, chronic pain (necessitating a neurospinal stimulator), and a prior history of sexual assault and intimate partner violence, who presented as a transfer from an outside institution due to concern for an intra-abdominal pregnancy and absent fetal cardiac activity. She was at approximately 30 weeks' gestation based on a prior 13-week ultrasound and had received markedly limited prenatal care during this pregnancy. Prior ultrasounds at that time had confirmed the presence of intrauterine pregnancy, with the most recent ultrasound evaluation occurring at about 24 weeks' gestation. She initially presented with worsening abdominal pain that was not resolved with over-the-counter medications. An ultrasound performed at an outside institution noted the presence of the fetus within the abdominal cavity and stated the placenta was adhered to the abdominal wall. The patient was readily transferred to our institution for further evaluation and care. Upon presentation, the initial vital signs were stable (Table 1).

**Table 1:** Patient physical examination values upon presentation

Vital Signs	Results	Reference Range
Blood pressure (mmHg)	117/75	90/60-120/80
Heart rate (beats per minute)	78	60-100
Temperature (F)	98.4	95.0-99.5
Respiratory rate (breaths per minute)	16	12-20
Pulse oximetry (%)	98	>92

**Figure 1:**
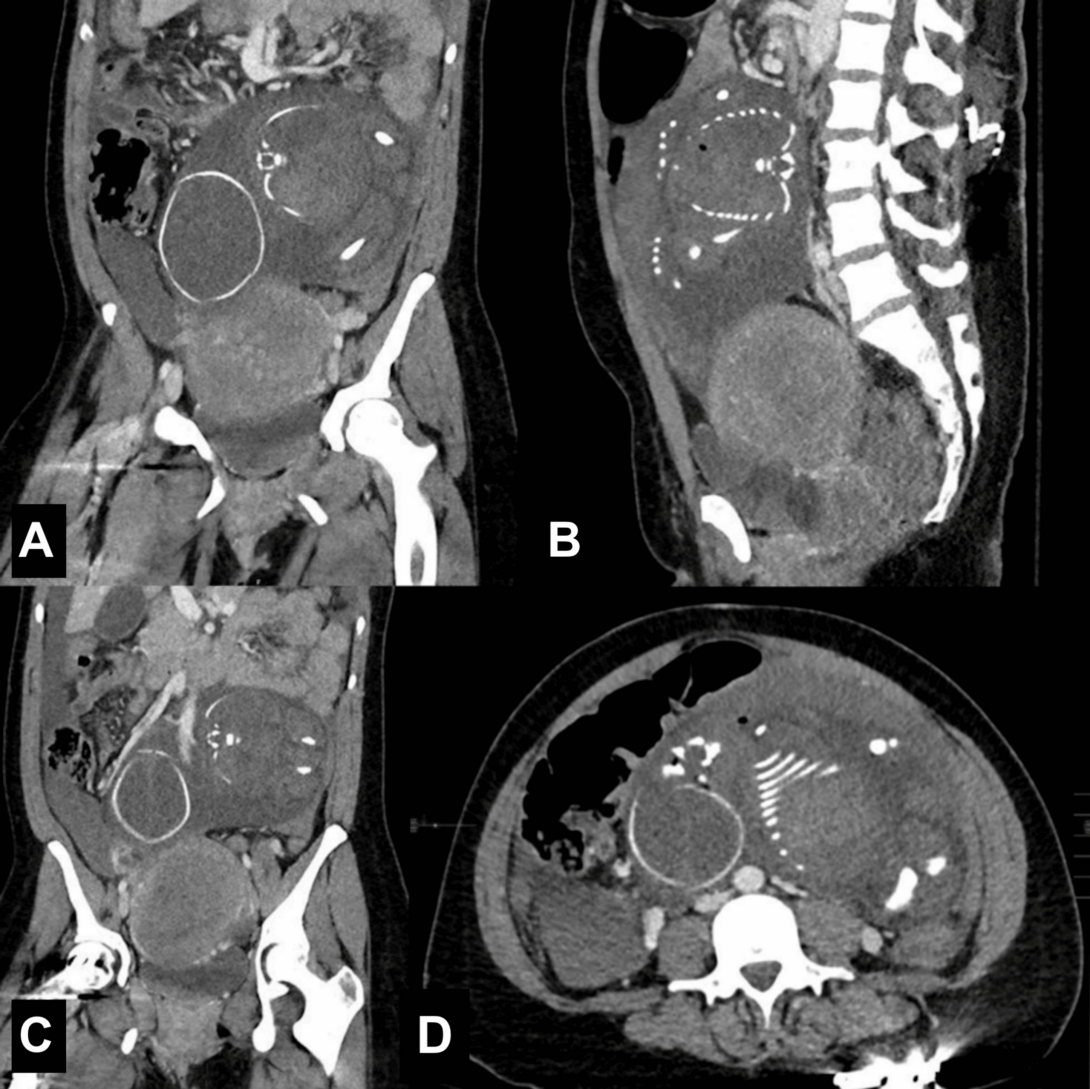
Computed tomography of the abdomen and pelvis with intravenous contrast. Coronal (A,C), sagittal (B), and axial views of the inferior abdomen and pelvis via computed tomography with intravenous contrast. Note the presence of a uterine defect along the right cornua (A). There are tiny foci noted within the thorax and neck of the fetus (B,D), with the presence of overlapping cranial bones (C,D).

The initial laboratory workup was notable for a critical hemoglobin level of 6.5 g/dL and leukocytosis of 16.8×10^3^/µL. Initial serum chemistries were unremarkable. A computed tomography (CT) scan of her abdomen and pelvis with intravenous contrast (Figure 1) demonstrated moderate volume hemoperitoneum, evidence of an intra-abdominal pregnancy along the superior aspect of the uterus, and an enlarged gravid uterus. A wedge-shaped defect along the superior right fundal region was also noted. There was a single fetus noted with overlapping cranial bones and a focus of air within the thorax and anterior neck, consistent with fetal demise.

**Figure 2:**
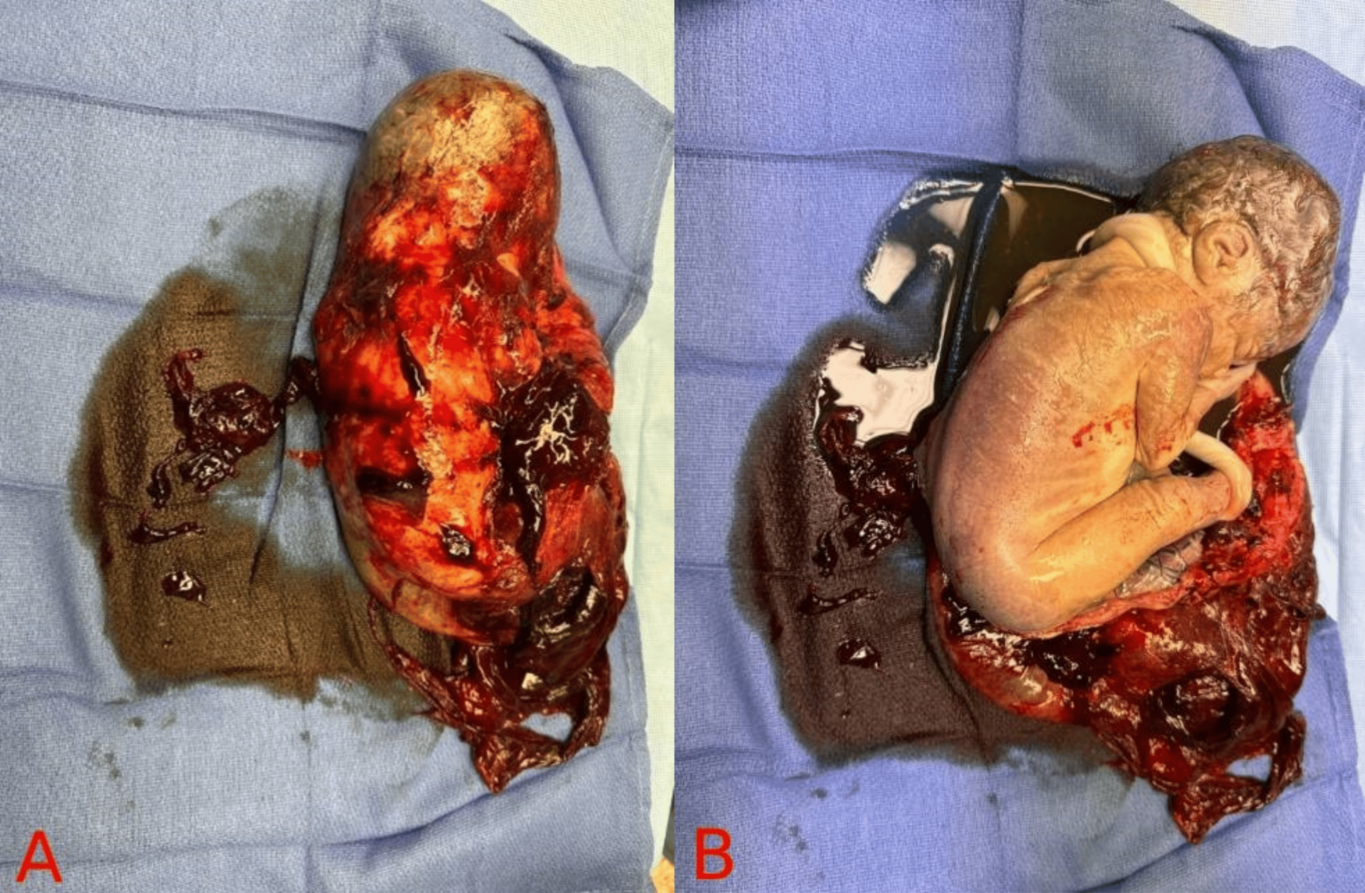
(A) En caul delivery of the intra-abdominal fetus. (B) Upon opening the amniotic sac, there is a developed non-viable female infant of approximately 30 weeks' gestation.

**Figure 3:**
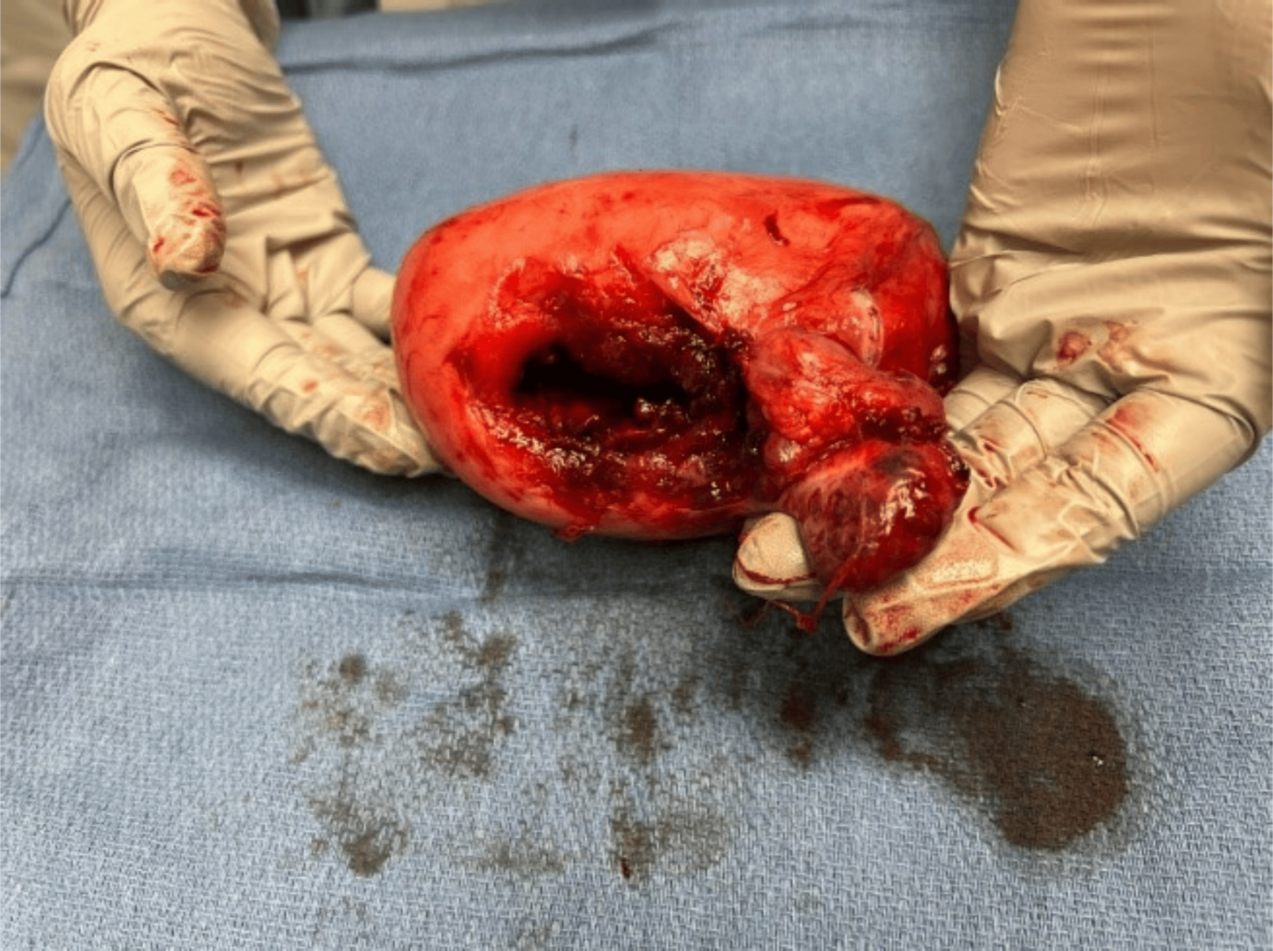
Uterine defect at the right cornua following subsequent hysterectomy.

The patient was taken to the operating room (OR) along with Obstetrics/Gynecology (OB/GYN) and General Surgery (GS) and underwent an exploratory laparotomy under general anesthesia. Upon entering the abdomen, a nonviable female infant was noted within the abdominal cavity, entirely outside of the uterus en caul, along with the placenta. The fetus was delivered by an OB/GYN (Figure 2). The patient was noted to have significant hemoperitoneum, with evacuation of approximately 1500 cc of blood products, necessitating transfusion of 5 units of packed red blood cells, 5 units of fresh frozen plasma, and 1 unit of platelets intraoperatively. Upon clearing the abdominal cavity of the initial hemoperitoneum, the uterus was evaluated and demonstrated an approximately 6 cm defect at the right cornua with evidence of active bleeding (Figure 3). Additionally, the right fallopian tube remnant and ovary were adhered to the right cornua, adjacent to the cornual defect/rupture site. The decision was made to proceed with a hysterectomy. Our patient recovered well postoperatively without any complications.

## Discussion

Spontaneous uterine rupture involves the complete rupture of the three layers of the uterus (endometrium, myometrium, and perimetrium) and is associated with significant morbidity and mortality for both mother and fetus [1,2]. Among the highest risk factors for uterine rupture are prior cesarean sections, multiparity, and prior uterine surgeries such as myomectomy and salpingectomy [1,3]. Over the last five decades, the rate of cesarean sections has increased by approximately 25% in the United States [1]. Efforts have been made to mitigate the associated risk of uterine rupture by decreasing the rate of cesarean sections [1].

The clinical presentation of uterine rupture can be variable, with the most crucial initial assessment being hemodynamic stability [1]. Therefore, it is imperative to note the importance of appropriate and timely radiologic imaging and correlation with appropriate labs in the setting of suspected uterine rupture. Initial ultrasound imaging for the patient was performed at an outside facility and demonstrated concerns for intra-abdominal pregnancy. Coupled with the patient's history and a critically low hemoglobin upon presentation, it raised concerns for possible uterine rupture and active hemorrhage. The absence of fetal heart tracings was also highly indicative of uterine rupture [1].

En caul describes the birth of the fetus and placenta within an unruptured amniotic sac. This is a relatively rare presentation, occurring approximately every one in 80,000 vaginal deliveries [5,6]. Delivery of an en caul fetus can occur naturally via vaginal birth or via cesarean delivery. Among the most significant risk factors are prematurity and low-gravida mothers. Literature reviews of an intraabdominal pregnancy "en caul" are exceedingly scarce, highlighting the rarity of this case. It is important to note that the patient had significant risk factors for spontaneous uterine rupture, given their history of previous ectopic pregnancies, cesarean sections, and prior salpingectomy [3]. There have been several case reports discussing uterine rupture following prior surgical interventions for ectopic pregnancies [2,3,6,9], notably prior dilation and curettage and prior salpingectomies, both with and without cornual resection.

According to the American College of Radiology (ACR) Appropriateness Criteria, a CT abdomen and pelvis with intravenous contrast may be an appropriate next step in evaluating the patient for signs of ongoing hemorrhage and identifying the source of bleeding [7]. It is normally ordered by the emergency medicine physician and obstetrician. The Committee Opinion of the American College of Obstetricians and Gynecologists (ACOG) is in accordance with ACR [7,8], stating that the use of CT and contrast material should not be withheld if clinically indicated. Given that the patient was hemodynamically stable at the time of presentation, the CT abdomen and pelvis provided critical information prior to the patient's subsequent surgery, most notably regarding the presence of active hemorrhage, location of the fetus, fetal viability status, uterine anatomy, and associated defects.

## Conclusions

Spontaneous uterine ruptures are rare but devastating complications that have a significant effect on maternal morbidity and mortality. They are associated with prior cesarean sections, multiparity, and other prior uterine surgeries. Initial symptoms can be nonspecific, such as generalized abdominal pain. Proper clinical evaluation, correlated with lab work and appropriate imaging in the emergency department setting, is crucial in establishing a potentially critical diagnosis before it worsens. In the setting of a suspected uterine rupture in the third trimester, a CT of the abdomen and pelvis with intravenous contrast is appropriate for evaluation and can provide highly valuable information, such as fetal viability, the presence of hemorrhage, and notable anatomical defects, prior to the patient arriving in the operating room.

## References

[1] Togioka BM, Tonismae T (2024). Uterine rupture. StatPearls [Internet].

[2] Košec V, Čukelj M, Djaković I, Butorac D (2021). Uterine rupture in third trimester of pregnancy following cornual resection due to ectopic pregnancy. Acta Clin Croat.

[3] Malik S, Fahad A (2023). Case of cornual uterine rupture in subsequent pregnancy following laparoscopic removal of cornual ectopic pregnancy. Cureus.

[4] Eboh S, Burghul S, Galloway M, Sanchez A, Ventolini G (2022). Preterm complete uterine rupture with en caul expulsion. Clin Med Insights Case Rep.

[5] Moran M (2022). Emergency department pre-viability delivery of a fetus en caul. Cureus.

[6] Pal A (2021). Twin caesarean delivery “en caul” - a case report and review of literature. J Evid Based Med Healthc.

[7] American College of Radiology (2020). ACR Appropriateness Criteria® Postpartum Hemorrhage. https://acsearch.acr.org/docs/3113019/Narrative/.

[8] American College of Obstetrics and Gynecology (2016). Guidelines for diagnostic imaging during pregnancy and lactation. Guidelines for Diagnostic Imaging During Pregnancy and Lactation.

[9] Pontis A, Prasciolu C, Litta P, Angioni S (2016). Uterine rupture in pregnancy: two case reports and review of literature. Clin Exp Obstet Gynecol.

